# Sequence and Expression Characteristics of Long Noncoding RNAs in Honey Bee Caste Development – Potential Novel Regulators for Transgressive Ovary Size

**DOI:** 10.1371/journal.pone.0078915

**Published:** 2013-10-31

**Authors:** Fernanda C. Humann, Gustavo J. Tiberio, Klaus Hartfelder

**Affiliations:** Departamento de Biologia Celular e Molecular e Bioagentes Patogênicos, Faculdade de Medicina de Ribeirão Preto, Universidade de São Paulo, Ribeirão Preto, São Paulo, Brazil; National Institutes of Health, United States of America

## Abstract

Division of labor in social insect colonies relies on a strong reproductive bias that favors queens. Although the ecological and evolutionary success attained through caste systems is well sketched out in terms of ultimate causes, the molecular and cellular underpinnings driving the development of caste phenotypes are still far from understood. Recent genomics approaches on honey bee developmental biology revealed a set of genes that are differentially expressed genes in larval ovaries and associated with transgressive ovary size in queens and massive cell death in workers. Amongst these, two contigs called special attention, both being over 200 bp in size and lacking apparent coding potential. Herein, we obtained their full cDNA sequences. These and their secondary structure characteristics placed in evidence that they are *bona fide* long noncoding RNAs (lncRNA) differentially expressed in larval ovaries, thus named *lncov1* and *lncov2*. Genomically, both map within a previously identified QTL on chromosome 11, associated with transgressive ovary size in honey bee workers. As *lncov1* was over-expressed in worker ovaries we focused on this gene. Real-time qPCR analysis on larval worker ovaries evidenced an expression peak coinciding with the onset of autophagic cell death. Cellular localization analysis through fluorescence *in situ* hybridization revealed perinuclear spots resembling omega speckles known to regulate trafficking of RNA-binding proteins. With only four lncRNAs known so far in honey bees, two expressed in the ovaries, these findings open a novel perspective on regulatory factors acting in the fine tuning of developmental processes underlying phenotypic plasticity related to social life histories.

## Introduction

Social organization and division of labor within a honey bee colony is characterized by marked physiological, behavioral and morphological differences between the queen and the workers, especially so in the reproductive system [1]. While a queen has almost 200 ovarioles in each of her ovaries and is capable of laying over 1000 eggs per day, the typical worker ovary consists of only 2–12 of these serial units [2], and while in the presence of the queen, workers normally do not lay eggs. Importantly, ovariole numbers are highly variable also within each of the castes, due to genetic factors, as well as nutritional conditions experienced during larval growth [3], [4], [5], [6] and there is also considerable variability in this character among subspecies of *Apis mellifera* and other species of the genus *Apis* [7]. Notwithstanding, while ovariole number is variable within each caste, the ovary phenotype of adult queens is separated from that of adult workers by a very large gap, thus presenting a clearly bimodal distribution. This bimodality is the result of a nutritionally determined massive programmed cell death occurring in the ovaries of worker larvae [8].

In the early larval stages, when worker larvae can still be shifted to develop as queens by transfer into queen cells, each of the ovaries is initially comprised of 150–200 ovariole primordia [9], but once a worker-destined larvae has molted into the last larval instar an onset of massive cell death is seen in her ovaries. This cell death, which shows characteristics of autophagy [10], starts in the germ cell region of the ovarioles and gradually expands towards the more apical and basal regions, so that by the end of the larval-pupal transition 90–99% of all the ovarioles are completely degenerated [11]. In contrast, in a queen-destined larva practically all of the ovariole primordia survive this critical period [10], [12], and this is due to a higher juvenile hormone titer in the hemolymph of queen larvae [13], [14], [15].

Aiming to understand the molecular underpinnings of this cell death program and, implicitely, the role and mode of action of juvenile hormone, we recently concluded an analysis of differential gene expression comparing early fifth instar queen and worker ovaries in a suppression-subtractive library (Representational Difference Analysis, RDA) experimental design [16]. When validating the expression through RT-qPCR assays of a set of differentially represented genes, our attention was drawn to two transcripts represented by several expression tags in the subtractive libraries, one being overexpressed in queen (Group11.35) and the other in worker ovaries (Group 11.31). The contigs consisting of two and five ESTs, respectively, were both over 200 bp in size but lacked an apparent coding potential. As these expression tags had not been computationally predicted as genes in the Official Gene set 2.0 for the honey bee [1], [17] we originally simply named them according to the genome scaffold they were located in, already considering that they might represent long noncoding RNAs [16].

Long noncoding RNAs (lncRNAs), a class of noncoding RNAs simply defined by molecular size (>200 nt), have drawn increased attention during the last decade, as in several of the genome sequencing and annotation projects they came to light as a class of genes that may by far exceed the number of protein coding genes [18]. But as they show little evolutionary conservation, they cannot easily be predicted by current genome annotation algorithms. Their location in the genome can be intronic to protein coding genes, or intergenic [19], [20]. Like conventional mRNAs, lncRNAs can be capped and polyadenylated, and while mostly derived from a single exon, several lncRNAs are known to be spliced [21], [22]. One frequently used criterion to distinguish noncoding from protein-coding RNAs is open reading frame (ORF) length, currently established at 100 amino acids (aa) as a threshold [23]. Nevertheless, there are also very long ncRNAs, such as *Xist*, one of the first members of this class to be recognized, that contains a putative ORF of 298 aa [24]. Furthermore, while there is generally little conservation in lncRNA nucleotide sequence across species, even between phylogenetically close ones, the lack of primary sequence conservation does not necessarily mean an absence of conservation in function [25], [26].

Functionally, lncRNAs are responsible for transcriptional gene silencing, either directly or through chromatin modification, changes in chromosome architecture, control of alternative pre-mRNA splicing, protein degradation, organelle biogenesis and subcellular trafficking (for reviews see [20], [26], [27], [28], [29]). In most of these cases, lncRNAs interact with proteins and/or DNA generating modular scaffolds for complex modulation and fine-tuning of cellular activity [30], [31].

In honey bees, only two lncRNAs had been identified prior to our work. These were a 17,525 nt nuclear RNA from the honey bee brain [32] and a 6,789 nt nuclear RNA, also expressed in brain tissue [33]. The complex functionalities of lncRNAs emergent in recent studies, primarily on mammalian model organisms, and the fact that we identified ESTs representing two putative new lncRNAs expressed in a developmental context that is crucial for establishing reproductive division of labor in a social insect [16], we herein describe their full length cDNA sequence, obtained through 3′5′ RACE, and their putative secondary structure. The gene evidenced as overexpressed in the ovary of worker larvae and mapping to the genomic scaffold Group 11.31 in the Honey Bee Genome assembly [1] version 4.0 was now renamed as long noncoding ovary-1 RNA *(lncov1)*, and the one over-expressed in queen ovaries and mapping to Group 11.35 as long noncoding ovary-2 RNA *(lncov2)*. Proper naming of these genes became necessary because, due to genomic gap sequencing and superscaffolding efforts, scaffold numbers are now reduced in the most recent version (4.5) of the honey bee genome assembly. *lncov1*, for instance, is now in Group 11.18 of chromosome 11. Since *lncov1* is expressed in the context of autophagic cell death we further focused on this gene and analyzed its temporal expression profile in larval worker ovaries, as well as its cellular localization by fluorescence *in situ* hybridization, so as to gain insights into possible functions.

## Results

### *lncov1* and *lncov2* sequence characteristics

**Figure 1:**
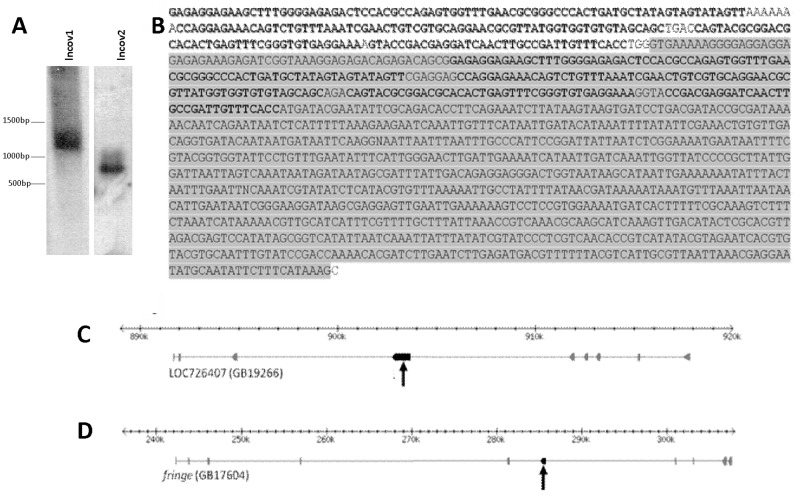
Full length cDNAs of honey bee *lncov1* and *lncov2* and their genomic mapping. (A) Southern Blot showing probe hybridizations with full-length cDNAs of *lncov1* and *lncov2* produced by 3′5′RACE reactions. The *lncov1* probe labelled a cDNA of 1367 bp, the one for *lncov2* hybridized to a 684 bp transcript. (B) Full length mRNA sequence of *lncov1*. Coverage with the genome sequence is shown on grey background, the tandem repeat sequence lacking in the genome is shown on white background. (C) Genomic mapping of *lncov1*; *lncov1* (arrow) is located in the sense strand to the fifth intron of the predicted protein LOC726407. (D) *lncov2* (arrow) maps into the fourth intron of *fringe*, also in sense direction.

As a first step we obtained full-length sequences for the two putative lncRNAs by means of a 3′5′RACE strategy. This and subsequent confirmation by Southern blot analysis (Figure 1A) showed that the full-length cDNA sequence of *lncov1* is 1367 bp (GenBank accession numbers JZ474541-JZ474546). In the honey bee genome assembly version 4.0, which was originally used for mapping the ESTs of our RDA study [16], it was located in scaffold Group 11.31. Remapping of the full-length *lncov1* cDNA to the genome sequence confirmed that it consists of a single exon. Furthermore, we found that *lncov1* has a tandem repeat of 250 bp in its 3′ region that is missing in the genome sequence, both in versions 4.0 and 4.5 (Figure 1B). The *lncov1* gene maps within the fifth intron of the coding strand of a predicted protein-coding gene (Figure 1C). This gene LOC726407, also named GB19266 in the Honey Bee Official Gene Set version 2.0, has eight exons, and has no function associated.

In our first annotation [16], *lncov1* was listed as a no-match sequence, but we could since find a sequence with significant similarity (E-value 1e^−25^) in the more recently deposited 454 transcriptome sequence reads generated from brain RNA of the stingless bee *Melipona quadrifasciata* [34], which is also a highly eusocial bee. Furthermore it had a perfect match (E-value 0.0) to a sequence in an *A. mellifera* shotgun transcriptome assembly (TSA) from embryonic and testis RNA. It is also highly similar (E-value 0.0) to a hypothetical miscRNA, LOC100578155, predicted in the most recent version of the *Apis mellifera* genome assembly (version 4.5). When conceptually translating the 1367 bp *lncov1* sequence, the longest ORF comprised only 23 amino acids. It had a TestCode analysis result of 0.407, with a high percentage of rare codons. A low coding potential score (−1.35548) returned by the Coding Potential Calculator software also placed in evidence that there is little probability for this ORF being a protein-coding sequence.

Similar characteristics were also denoted for *lncov2* which had been evidenced as over-expressed in queen ovaries. Sequencing of the 3′5′RACE products and Southern blot analysis (Figure 1A) resulted in a coding sequence of 684 bp (GenBank accession numbers JZ474547–JZ474553) that perfectly matched within the genome scaffold Group 11.35 of the honey bee genome assembly version 4.0, and in Group 11.18 of version 4.5, respectively. Furthermore, this re-mapping revealed that the *lncov2* transcript is derived from two exons, a small first exon of only 41 bp and a larger second one of 643 bp. The *lncov2* gene is an intronic sequence located within the fourth intron of the gene *fringe*, given as GB17604 in the Official Gene set 2.0 (Figure 1D). While originally also reported as a no-match sequence [16], transcripts similar to honey bee *lncov2* could be found in the more recently released *A. mellifera* TSA database. A TestCode analysis run on *lncov2* showed that this transcript also has a very low probability of encoding a protein (0.446). This is also corroborated by the Coding Potential Calculator results, which attributed a 35% coding potential probability, meaning a weak noncoding potential (−0.985348).

**Figure 2:**
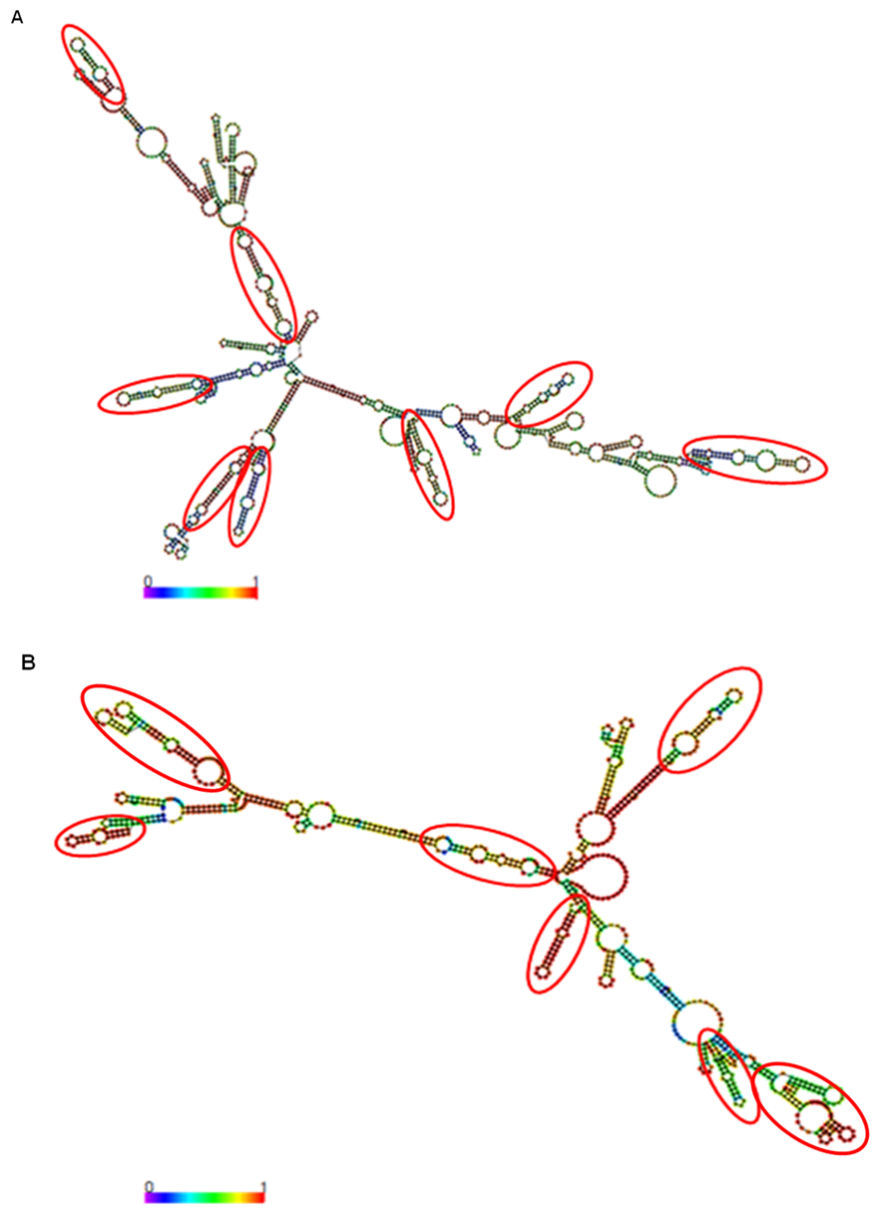
Secondary structure predictions based on minimum free energy of (A) *lncov1* and (B) *lncov2* RNA. The color scale indicates high (red) to low (blue) probabilities of base pairing. Circles indicate conserved miRNA/hairpin structures present in the secondary structure of the long noncoding RNAs. Secondary structure predictions were made using RNAfold software.

So as to obtain further evidence for the classification of *lncov1* and *lncov2* as lncRNAs we performed secondary structure analyses by means of the RNAfold program. This showed that both RNAs have series of hairpin consensus structures with high base pairing probabilities (Figure 2). Minimum free energy was calculated as −361.96 kcal/mol for *lncov1* and −271.79 kcal/mol for *lncov2*. These findings further corroborate the putative classification of the two differentially expressed noncoding RNAs from the RDA libraries of honey bee ovaries as *bona fide* intronic lncRNAs.

### Genomic localization of *lncov1* and *lncov2* within a QTL for ovary size variation

**Figure 3:**
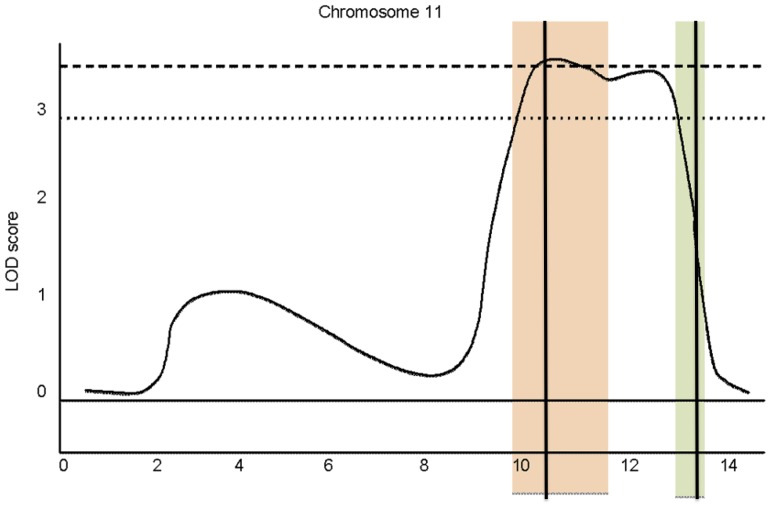
Schematic representation of the *lncov1* and *lncov2* gene positions in the chromosome 11 region containing a QTL for transgressive ovary size in workers. The dotted and dashed LOD score lines represent the 95% and 99% QTL thresholds, respectively, above which ovariole number is significantly influenced [4], [6]. The orange bar indicates the location of Group 11.31 scaffold and the green one the Group 11.35 scaffold (genome version 4.0). Black vertical lines indicate the respective positions of *lncov1* and *lncov2* within these scaffolds.

The fact that *lncov1* and *lncov2* both map to a relatively narrow genomic region on chromosome 11 made us take a closer look at this region, and a fortuitous finding was the report of a Quantitative Trait Locus (QTL) for variation in ovariole number of honey bee workers [6], [35]. This QTL on chromosome 11 was identified in backcrosses of Africanized with European honey bees, which exhibit different worker ovariole phenotypes. The QTL is above the 99% genome-wide threshold, with a 95% confidence interval covering the map interval between positions 8.9 and 12.2 Mb on chromosome 11. Strikingly, a genome browser analysis in BeeBase revealed that *lncov1* maps right in the center of this interval, while *lncov2* is located close to the 12.2 Mb border of the QTL (Figure 3).

### Expression and cellular localization of *lncov1*

**Figure 4:**
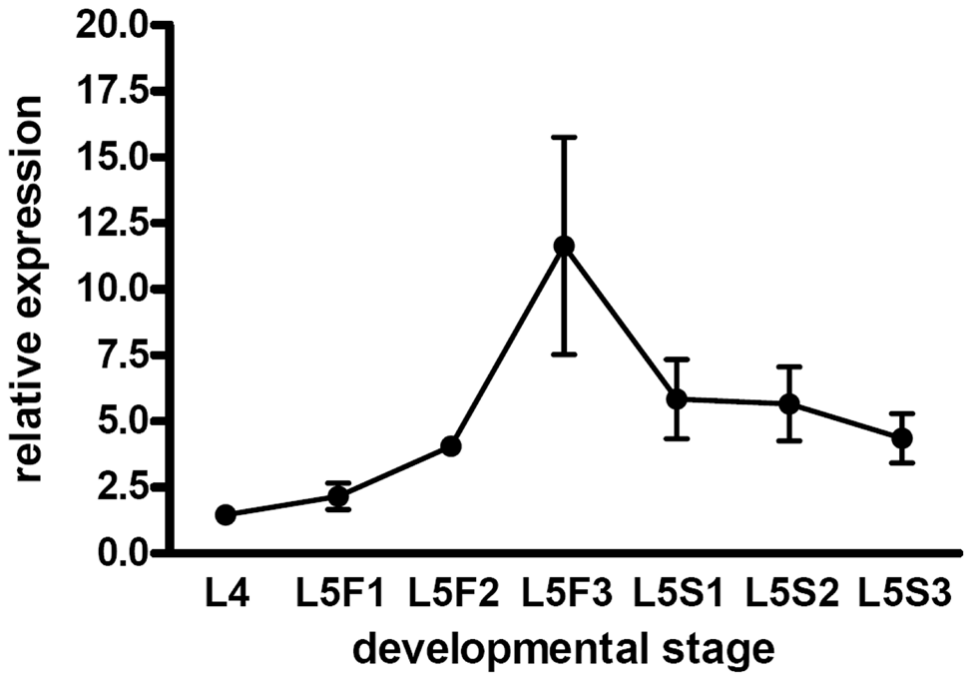
Relative expression (2^−ΔΔCt^) of *lncov1* in ovaries of worker larvae, spanning the fourth (L4) and the feeding (L5F1–L5F3) and spinning stages (L5S1–L5S3) of the last larval instar. A peak in *lncov1* transcript abundance is apparent in the L5F3 stage, when autophagic cell death becomes a prominent feature in worker ovaries [14].

To gain further insights into the functionality of the lncRNAs we decided to focus on *lncov1* because it is overexpressed in larval worker ovaries undergoing autophagic cell death, has a strong noncoding potential, and is located at a central position within the above mentioned QTL. As a first step we analyzed the relative expression levels of *lncov1* by real-time qPCR in ovaries of worker larvae, covering the critical period for caste differentiation in the fourth and the fifth larval instars [3]. The results shown in Figure 4 revealed a gradual increase in ovarian *lncov1* transcript levels from the fourth instar (L4) through the early feeding phases of the fifth instar (L5F1 and L5F2) and a marked peak of expression at the end of the feeding stage (L5F3). Subsequently, as the brood cells are sealed and the larvae start to spin their cocoon and enter metamorphosis, the *lncov1* transcript levels drop again and remain at a level similar to the one attained in L5F2. This finding is supporting evidence for a gene function in a temporal window when autophagic cell death is the main cellular event seen in the ovaries of honey bee worker larvae.

So as to further investigate this hypothesis, and in the absence of high resolution functional genomics assays for honey bee lncRNAs, we considered that the intracellular localization of *lncov1* could provide further hints and serve as a proxy for functionality because many noncoding RNAs exhibit certain specificity with respect to nuclear or cytoplasmatic localization [36]. To this end we performed fluorescent *in situ* hybridization (FISH) assays with an *lncov1* probe on whole mounts of ovaries dissected from early fifth instar worker larvae, this being the developmental stage used for generating the RDA libraries [16].

**Figure 5:**
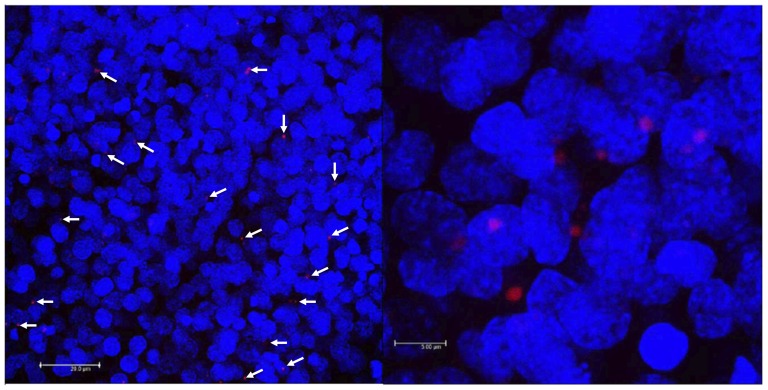
Fluorescence *in situ* hybridization (FISH) revealing the localization of *lncov1* transcripts in ovaries of fifth instar worker larvae. (A) Detection of an Alexa 594-labeled *lncov1* probe (red) in ovary whole mounts; nuclei are counterstained with DAPI (blue). White arrows indicate some of the *lncov1* agglomerates, showing their distribution throughout the developing ovarioles. (B) High resolution image of (A) evidencing *lncov1* RNA agglomerates in perinuclear positions. The images were captured by confocal microscopy. Scale bars represent 20 and 5 µm, respectively.

The FISH assays revealed distinct focal spots of *lncov1* RNA (Figure 5). Through comparison with DAPI stained nuclei in the single image series of optical 0.5 µm sections, these *lncov1* spots turned out to be of primarily cytoplasmic localization. Their perinuclear localization is indicative of an omega speckle-like structure.

## Discussion

Two transcript fragments previously detected in the context of ovary differentiation of honey bee larvae and predicted as potential lncRNAs [16], were herein further investigated. After obtaining their full length cDNA sequences by means of a 3′5′RACE strategy and estimating transcript size by Southern blotting, the bioinformatics analyses of their primary and secondary RNA structure confirmed their low coding potential and revealed hairpin consensus structures, both typical characteristics of noncoding RNAs. Thus, *lncov1* and *lncov2* can be considered *bona fid*e honey bee lncRNAs.

Prior to this work, only two lncRNAs had been described in honey bees [32], [33], [37], these being expressed in the brain of adult bees. Hence, with only four lncRNAs identified so far, the analysis of this class of transcripts has not even reached infancy in the honey bee, this standing in utter contrast with knowledge on honey bees microRNAs, and even more so with the human transcriptome where lncRNAs now comprise the largest fraction amongst noncoding RNAs [38]. MicroRNAs have come to attention during the honey bee genome sequencing project. Several members were computationally predicted [1], [39] and their expression confirmed in large-scale screens [40], [41]. In contrast, information on long noncoding RNAs is extremely rare in this and also other social insects.

Small RNAs can not only be computationally predicted in sequenced genomes, they can also be selectively sequenced by RNAseq technology [42]. But this is not the case for lncRNAs. Due to a lack in sequence conservation they cannot be *ab initio* predicted in genome assemblies, and when massively coming to light in tiling arrays and RNAseq, their classification as lncRNAs still requires in-depth sequence-by-sequence analysis. The primary criterion used to distinguish lncRNAs from protein-coding ones is ORF length, with a cutoff value set at 100 codons by the FANTOM consortium [43]. This criterion alone is, however, insufficient to define a putative lncRNA as such. Calculation of coding potential and secondary structure analysis revealing conserved miRNA functional motifs as domains within an lncRNA, as achieved through the use of Coding Potential Calculator and RNAfold softwares, are good additional evidence. Nonetheless, functional ncRNAs may contain secondary or tertiary structures with non-canonical base interactions [44] that are not detected by such programs.

Despite these difficulties we nevertheless expect to see an exponential increase in lncRNAs being identified in the honey bee genome, as even in small subtractive libraries, specifically designed and directed towards detecting differential gene expression in honey bees, substantial numbers of transcripts are frequently classified as no-match sequences [16], [45]. For obvious reasons, RNAseq libraries and EST databases for bees have so far mainly been searched for predicted gene models [46], leaving data mining for lncRNAs as a yet unexplored bonanza. The finding that both *lncov1* and *lncov2* are located within introns of predicted genes characterizes them as intronic lncRNAs, most of which are located within the first introns of their host genes, with a strongly 5′-biased positional distribution [47].

In *Drosophila melanogaster*, the first evidence for lncRNAs appeared only in the late 1980's, when Rao and colleagues [48] suggested that the 3′-end-transcribed but untranslated region of *act5C* could be involved in regulating actin gene expression. Since that date, hundreds of putative lncRNAs have been identified in high throughput transcriptome analyses, but as yet only few of these have any function associated.

lncRNAs have the ability to act in *trans* (on distantly located genes) or in *cis* (on neighboring genes), and the latter may especially be the case for the relationship between intronic lncRNAs and their genomic host genes. While little can be said in this regard about the host gene for *lncov1*, LOC726407, the host gene for *lncov2* is predicted as *fringe*, an important developmental gene in *Drosophila*. This homolog to vertebrate *lunatic fringe* is involved in compound eye development, wing and leg disc patterning, egg-chamber formation and several other processes, and it exerts this role primarily through regulating Notch signalling. Interestingly, the QTL identified on chromosome 11 also contains a honey bee Notch ortholog (LOC410351) [6]. As we found *lncov2* to be overexpressed in larval queen ovaries, this lncRNA and its host gene may be involved in the JH-dependent maintenance of the developing ovarioles in the early fifth instar of queen larvae. In this stage these show considerable elongation and the terminal filament and germarial region become clearly structured [10]. Obviously, this hypothesis needs to be addressed in expression and functional assays for *lncov2* and its host gene.

As in the current study we were more interested in *lncov1*, we assayed its temporal expression pattern in worker larval ovaries covering the phase when cell death becomes a major of factor in shaping adult ovary size [10], [11], [12]. The relative transcript abundance of *lncov1* reached peak levels in the fifth instar, right at the time when these larvae are growing most and reach the final weight before entering metamorphosis. This temporal coincidence of *lncov1* expression with the main period of autophagic cell death in the larval worker ovary is strongly suggestive that it is functionally involved in this process.

To gain first insights into its functionality and, especially so, to get an idea on whether it might be a *cis* or *trans* acting lncRNA, we investigated its cellular localization by means of a FISH assay. Intronic lncRNAs are generally spatially restricted to nuclear or cytoplasmatic cellular extracts [49], and their expression is frequently related to responses to environmental modification [50], [51]. We observed that *lncov1* mRNA was concentrated in punctate agglomerates near the nuclei of ovarian cells, and seemingly this was generally in a single spot-like structure. Although this may be indicative of a *trans*-acting function of *lncov1*, possibly as a component of a ribonucleoprotein (RNP) complex involved in processing other RNAs, its specific function still remains to be identified. Interestingly, a similar localization pattern has been observed for the heat shock RNA omega (*hsrω*) noncoding gene in *Drosophila melanogaster* which forms nucleoplasmic omega speckles [52]. These structures act as dynamic sinks that regulate the trafficking of heterogeneous nuclear RNA-binding proteins (hnRNPs) and of other related RNA-binding proteins. When silencing the *hsrω* gene function by means of an RNAi experiment, Mallik and Lakhotia [52] found an inhibition of the apoptosis response to stress condition, and a subsequent study [53] showed that *hsrω* genetically interacts with the *Drosophila* chromatin remodelling ATPase ISWI, bringing to light a direct connection between chromatin remodelling, omega speckles and cellular responses to stress. Consequently, the stress-related lncRNAs are thought to function as hubs, bringing together networks of protein-RNA interactions that maintain the intricate balance in cellular homeostasis [54].

Although at present the connection between *lncov1* localization in honey bee ovaries and omega speckles in *Drosophila* is only speculative, requiring colocalization analysis of putative omega speckle components and *lncov1* RNA in honey bee ovaries, the parallels between the two biological systems are striking. Both are related to stress responses and integrate with epigenetic regulation. In honey bee larvae, the expression analysis of core genes of the hypoxia signalling pathway revealed higher relative transcript levels of the genes encoding HIF1α, HIF1β and PHD homologs in worker larvae, with an expression peak in the early fifth instar larvae [55]. This endogenous hypoxia response due to diminished mitochondrial activity in worker larvae could be connected with the lower sugar concentrations in worker larval diet [56]. Acting as environmental (nutritional) trigger this is associated, on the one hand, with lower JH titers in worker larvae [13], and on the other with *de novo* DNA methylation in these larvae. When silencing the DNA methyltransferase 3 encoding gene in honey bee larvae by means of an RNAi approach, Kucharski *et al.* [57] found that the resultant adults had a more queen-like phenotype, especially with respect to ovary size. This network of environmental, endocrine and genetic/epigenetic influences driving caste development in honey bees has recently been brought together in a mathematical model, with ovary size as the readout [8].

Variation in ovary size, *viz.* ovariole number, within insect species is not uncommon [58] and is currently best understood in *Drosophila melanogaster* [59]. In flies, a *bric-a-brac-*dependent cell fate determination sets up a terminal filament structure [60], which then serves as an anchor for the apical-to-basal progression of a basal lamina segregating columns of germline and somatic cells into ovariole primordia [61]. Rearing conditions during the larval stages, especially so nutritional quality, have recently been shown to affect terminal filament cell number and size through the insulin-insulin-like/target-of-rapamycin (IIS/TOR) and Hippo signalling pathways, hence resulting in intra and interspecific variation in ovariole number in fruit flies [62].

The difference between the worker and the queen ovary phenotype in bees is, however, not contingent on differences in terminal filament organization, as similar numbers of ovariole primordia are initially formed in both castes [9], [10] and preceding environmentally induced caste determination. Rather, the developmental commitment to a queen or worker ovary phenotype initiates in the third larval instar [3], and the onset of programmed autophagic cell death in the early fifth instar is accompanied by a disruption in the organization of the actin cytoskeleton in the rosettes of germline cells [15]. Thus, certain genes revealed as differentially expressed in this developmental phase may be directly involved in the developmental processes shaping the respective ovary phenotypes of queens and workers. The two long noncoding RNAs identified in the RDA screen [16] and characterized herein in terms of gene structure and expression characteristics, are, thus, particularly attractive for further in-depth studies. They are the first long noncoding RNAs identified in honey bees associated with a biological context that is highly relevant for reproductive division of labor in an insect society.

## Materials and Methods

### Honey bee larvae

Batches of fourth and fifth instar worker larvae were retrieved from brood combs of hives of Africanized hybrid bees (*Apis mellifera*) kept in the Experimental Apiary of the University of São Paulo, Ribeirão Preto, Brazil. Staging was done following established criteria [13], [63]. The larvae were immediately dissected in cold Ringer solution (85.5 mM NaCl, 5.6 mM KCl, 1.7 mM CaCl_2_x2H_2_O) and the ovaries removed and cleaned from adhering fat body.

For 3′5′RACE and RT-qPCR, batches of 20 pairs of ovaries were transferred to 1 mL of Trizol reagent (Invitrogen) and frozen at −80°C for subsequent extraction of total RNA. Extracted RNA was treated with 0.1 U DNaseI (Invitrogen) and RNA quality and quantity were assessed spectrophotometrically. First strand cDNA was produced using a SuperScript II (Invitrogen) protocol at 42°C for 50 min and 70°C for 15 min.

For *in situ* hybridization the dissected ovaries were immediately fixed in a heptane/paraformaldehyde mix [82% heptane, 13.12%, HEPES buffer (0.1 M HEPES, pH 6,9; 2 mM MgSO4; 1 mM EGTA), 0.66% paraformaldehyde, 1.64% DMSO], washed with methanol and twice with ethanol 100%, and stored in ethanol at 20°C until use.

### 3′5′ RACE and Southern blot analysis

The RACE reactions were performed using a Marathon cDNA Amplification kit (Clontech) protocol, with priming reaction temperature set at 68°C. For *lncov1*, the following primers were designed: OFctg17RACE 5′-GAGAGGAGAAGCTTTGGGGAGAGAC-3′ and ORctg17RACE 5′-GCTGCTACACACCACCATAACGC-3′; for *lncov2* these were RFctg9RACE 5′-CGAAGATAAACAGGACCGACACC-3′ and RRctg9RACE 5′-GAAGAACGACGAAAAGTTGAGCGG-3′.

The RACE reaction products were electrophoresed in 1,2% agarose gels, visualized by ethidium bromide staining, and blotted by capillarity onto a Hybond-N nylon membrane (GE Healthcare). Probe labelling and hybridization were done according to the Amersham Gene Images AlkPhos Direct Labelling and Detection System (GE Healthcare) protocol. The probe for *lncov1* (146 bp in length) was produced by PCR with the following primers: Ocont17F 5′-GGAGAAGCTTTGGGGAGAG-3′ and Ocont17R:5′-CTGCTACACACCACCATAAC-3′. The probe for *lncov2* was produced with the primers Rcont9F: 5′-CGAAGATAAACAGGACCGAC-3′ and Rcont9R: 5′-AGAAGGAAGTGAATTGAAGAAC-3′, resulting in a product of 150 bp. Both probes were purified with Illustra GFX PCR DNA and Gel Band Purification kits (GE Healthcare).

Sequences corresponding to 3′ and 5′-ends and the overlapping region of *lncov1* and *lncov2*, were ligated into pGEM-T Easy Vector (Promega) and used to transform *E. coli* DH5α chemocompetent cells. The inserts were purified by a miniprep protocol and sequenced using the Big Dye Terminator Cycle Sequencing Ready Reaction (Applied Biosystems) with M13 primers on an ABI-PRISM 3100 (Applied Biosystems) automated gene analyzer.

### Bioinformatics analysis

After sequence quality analysis (PHRED-PHRAP implemented in the E-Gene platform, [64]) vector sequences were trimmed and the obtained sequences were assembled using CAP3 [65] to obtain a complete sequence for each transcript. These were then mapped to assembly version 4.0 of the *Apis mellifera* genome [1] using Artemis v. 7.0 genome annotation software [66] implemented on a Linux server.

The secondary structures with minimum free energy for *lncov1* and *lncov2* transcripts were analyzed and visualized online using tools of the RNAfold program (http://rna.tbi.univie.ac.at) [67]. The intronic position of the *lncov1* and *lncov2* genes in their respective host genes, as well as their position within a major QTL for variation in worker ovariole number [6], [35], was analyzed using the Genome Browser implemented in Beebase (http://hymenopteragenome.org/beebase/). Rare codons usage was checked by Testcode analysis [68] (http://gcat.davidson.edu/DGPB/testcode.html) and potential coding capacity was estimated Coding Potential Calculator [69] (http://cpc.cbi.pku.edu.cn/).

### Real Time quantitative PCR

Specific primers previously designed and validated for *lncov1* [16] were used for the quantification of transcript levels. A SYBR Green protocol was run in an ABI-PRISM 7500 Real-Time PCR System (Applied Biosystems). The reactions consisted of 1X SYBR Green mix (Maxima SYBR Green qPCR Master Mix, Fermentas), 10 µM of each of the primers F and R, 1 µl of cDNA and 4 µl of sterile water completing a total volume of 14 µl. The reactions were performed under the following conditions: 50°C for 2 min, 95°C for 10 min, 40 cycles at 95°C for 15 s and 60°C for 1 min. Fluorescence readings were always taken at this last step.

After finishing the reaction cycles, the specificity of the products was checked by running a dissociation curve analysis protocol, starting at 95°C for 15 s, 60°C for 1 min and 95°C for 15 s. Data were collected in the last two steps. Criteria for primer quality were a single peak of dissociation and amplification efficiencies between 80–110%.

The quantification of *lncov1* transcript levels was done on cDNA samples of ovaries dissected from worker larvae in the fourth instar (L4), and the substages of the fifth instar covering the feeding (L5F1–L5F3) and the cocoon spinning phases (L5S1–L5S3). Three independent biological samples were prepared for each stage, each consisting of 15 pairs of ovaries. Each biological sample was analyzed in technical triplicates. Transcript abundance for *lncov1* and the reference gene *actin*, validated for RT-qPCR assays in honey bees [70], were expressed as Ct values (threshold cycle). Relative expression levels of *lncov1* were expressed as 2^−ΔΔCt^ [71] in relation to L4 transcript levels.

For the statistical analysis of expression, the 2^−ΔΔ^Ct values for the developmental stages were log transformed and analyzed with a Kruskal-Wallis test followed by *post hoc* Dunn's Multiple Comparison testing, these implemented in GraphPad Prism v 4.0. The level of significance considered was P≤0.05.

### Fluorescence *in situ* hybridization (FISH)

*In situ* localization of *lncov1* transcripts was analyzed in ovaries of worker larvae in the late feeding stage (L5F3). Antisense and sense probes were synthesized using *lncov1* specific primers containing a T7 promoter sequence (underlined) at the respective 5′ends (11.31FT7 5′-TAATACGACTCACTATAGGGCTGAGTTTCGGGTGTGTGAGG-3′; 11.31RT7 5′-TAATACGACTCACTATAGGGCTGAGTTTCGGGTGTGAGG-3′) in combination with the corresponding primers lacking this T7 sequence (11.31F and 11.31R). Amplification parameters were 94°C for 2 min, 40 cycles of 94°C for 45 s, 60°C for 45 s, 72°C for 1 min, and a final extension step of 72°C for 10 min. The fragments generated for the 11.31 primer combinations (11.31FT7+11.31R and 11.31RT7+11.31F) had a product length of 151 bp. The amplification products were checked in an agarose gel, purified (Illustra GFX PCR DNA and Gel Band Purification kit, GE Healthcare) and quantified spectrophotometrically. Subsequently, RNA probes were generated from these products by *in vitro* transcription from the T7 promoter using a FISH Tag RNA kit (Invitrogen).

The ovaries were processed following the fluorescent *in situ* hybridization procedure described for *Drosophila melanogaster* ovaries [72]. Briefly, the fixed ovaries were hydrated first in methanol, then with a mixture of methanol/PTw (PBS 1%, Tween 0.1%), and finally 3 X with PTw. The material was transferred to a DMSO 1∶9 PPTwT solution (PTw, paraformaldehyde 4%, Triton ×100 0,1%) for 20 min at room temperature. After five washes in PTw, they were incubated for 30 s with protease K (40 µg/mL) and again washed with glycine (10 mg/mL) in PTw. Following two washes with PTw they were re-fixed with PPTwT and washed 5× in PTw. Before hybridization, the ovaries were incubated for 10 min in PTw/HS (50% formamide, 4X SSC, 50 µg/mL heparin, 1X Denhardt's solution, 250 µg/mL yeast RNA, 500 µg/mL salmon testes DNA), and another 10 min with HS alone. After 1 h at 45°C in new HS the fluorescent RNA probe was added and hybridization proceeded for 16 h at 45°C. The RNA probe had been synthesized with the FISH Tag RNA kit (Invitrogen), following the manufacturer's instructions. Subsequently, the labeled ovaries were washed twice with HS and sequentially with HS/PTw (3∶1), HS/PTw (1∶1), HS/PTw (1∶3) and PTw. Nuclei were labeled with DAPI/PTw (4000∶1) and images of 0.5 or 1 µm optical sections were captured by laser confocal microscopy in a TCS-SP5 System (Leica). Leica LAS-AF software was used for image acquisition and processing. No adjustments were made with respect to image brightness and/or contrast.

## References

[1] The Honey Bee Genome Sequencing Consortium (2006) Insights into social insects from the genome of the honeybee *Apis mellifera* . Nature 443: 931–949. 17073008 10.1038/nature05260 PMC2048586

[2] Snodgras RE (1956) Anatomy of the Honey Bee. London: Comstock Publishing Associates. 334 p.

[3] Dedej S, Hartfelder K, Aumeier P, Rosenkranz P, Engels W (1998) Caste determination is a sequential process: effect of larval age at grafting on ovariole number, hind leg size and cephalic volatiles in the honey bee (*Apis mellifera carnica*). J Apic Res 37: 183–190.

[4] Linksvayer TA, Kaftanoglu O, Akyol E, Blatch S, Amdam GV, et al (2011) Larval and nurse worker control of developmental plasticity and the evolution of honey bee queen-worker dimorphism. J Evol Biol 24: 1939–1948. 21696476 10.1111/j.1420-9101.2011.02331.x PMC3156273

[5] Makert GR, Paxton RJ, Hartfelder K (2006) Ovariole number - a predictor of differential reproductive success among worker subfamilies in queenless honeybee (*Apis mellifera* L.) colonies. Behav Ecol Sociobiol 60: 815–825.

[6] Graham AM, Munday MD, Kaftanoglu O, Page Jr RE, Amdam GV, et al (2011) Support for the reproductive ground plan hypothesis of social evolution and major QTL for ovary traits of Africanized worker honey bees (Apis mellifera L.). BMC Evol Biol 11: e95. 10.1186/1471-2148-11-95 PMC3100260 21489230

[7] Hodin J (2009) She shapes events as they come: Plasticity in female insect reproduction. In: Whitman D, Ananthakrishnan TN, editors. Phenotypic plasticity of insects: mechanisms and consequence. Enfield, NH, USA: Science Publishers. pp. 423–521.

[8] Leimar O, Hartfelder K, Laubichler M, Page Jr RE (2012) Development and evolution of caste dimorphism in honeybees - a modelling approach. Ecol Evol 3: e414. 10.1002/ece3.414 PMC3539003 23301175

[9] Zander E, Löschel F, Meier K (1916) Die Ausbildung des Geschlechtes bei der Honigbiene (*Apis mellifica* L.). Z Angew Entomol 3: 1–74.

[10] Hartfelder K, Steinbrück G (1997) Germ cell cluster formation and cell death are alternatives in caste-specific differentiation of the larval honey bee ovary. Invertebr Reprod Dev 31: 237–250.

[11] Schmidt Capella IC, Hartfelder K (1998) Juvenile hormone effect on DNA synthesis and apoptosis in caste-specific differentiation of the larval honey bee (*Apis mellifera* L.) ovary. J Insect Physiol 44: 385–391. 12770156 10.1016/s0022-1910(98)00027-4

[12] Reginato RD, da Cruz-Landim C (2002) Morphological characterization of cell death during the ovary differentiation in worker honey bee. Cell Biol Int 26: 243–251. 11991652 10.1006/cbir.2001.0839

[13] Rachinsky A, Strambi C, Strambi A, Hartfelder K (1990) Caste and metamorphosis - hemolymph titers of juvenile hormone and ecdysteroids in last instar honeybee larvae. Gen Comp Endocrinol 79: 31–38. 2354779 10.1016/0016-6480(90)90085-z

[14] Rembold H (1987) Caste specific modulation of juvenile hormone titers in *Apis mellifera* . Insect Biochem 17: 1003–1006.

[15] Schmidt Capella IC, Hartfelder K (2002) Juvenile-hormone-dependent interaction of actin and spectrin is crucial for polymorphic differentiation of the larval honey bee ovary. Cell Tissue Res 307: 265–272. 11845333 10.1007/s00441-001-0490-y

[16] Humann FC, Hartfelder K (2011) Representational Difference Analysis (RDA) reveals differential expression of conserved as well as novel genes during caste-specific development of the honey bee (*Apis mellifera* L.) ovary. Insect Biochem Mol Biol 41: 602–612. 21477651 10.1016/j.ibmb.2011.03.013

[17] Elsik CG, Worley KC, Zhang L, Milshina NV, Jiang H, et al (2006) Community annotation: procedures, protocols, and supporting tools. Genome Res 16: 1329–1333. 17065605 10.1101/gr.5580606

[18] Mattick JS (2011) The double life of RNA. Biochimie 93: 8–9. 10.1016/S0300-9084(11)00355-5 21963144

[19] Guttman M, Donaghey J, Carey BW, Garber M, Grenier JK, et al (2011) lincRNAs act in the circuitry controlling pluripotency and differentiation. Nature 477: 295–260. 21874018 10.1038/nature10398 PMC3175327

[20] Louro R, Smirnova AS, Verjovski-Almeida S (2009) Long intronic noncoding RNA transcription: expression noise or expression choice? Genomics 93: 291–298. 19071207 10.1016/j.ygeno.2008.11.009

[21] Mercer TR, Dinger ME, Mattick JS (2009) Long non-coding RNAs: insights into functions. Nat Rev Genet 10: 155–159. 19188922 10.1038/nrg2521

[22] Wilusz JE, Sunwoo H, Spector DL (2009) Long noncoding RNAs: functional surprises from the RNA world. Genes Dev 23: 1494–1504. 19571179 10.1101/gad.1800909 PMC3152381

[23] Maeda N, Kasukawa T, Oyama R, Gough J, Frith M, et al (2006) Transcript annotation in FANTOM3: mouse gene catalog based on physical cDNAs. PLoS Genetics 2: 498–503. 10.1371/journal.pgen.0020062 PMC1449903 16683036

[24] Brockdorff N, Ashworth A, Kay GF, McCabe VM, Norris DP, et al (1992) The product of the mouse *Xist* gene is a 15 kb inactive X-specific transcript containing no conserved ORF and located in the nucleus. Cell 71: 515–526. 1423610 10.1016/0092-8674(92)90519-i

[25] Pang KC, Frith MC, Mattick JS (2006) Rapid evolution of noncoding RNAs: lack of conservation does not mean lack of function. Trends Genet 22: 1–5. 16290135 10.1016/j.tig.2005.10.003

[26] Ponting CP, Oliver PL, Reik W (2009) Evolution and functions of long noncoding RNAs. Cell 136: 629–641. 19239885 10.1016/j.cell.2009.02.006

[27] Ma H, Hao Y, Dong X, Gong Q, Chen J, et al (2012) Molecular mechanisms and function prediction of long noncoding RNA. Scient World J 2012: e541786. 10.1100/2012/541786 PMC3540756 23319885

[28] Malecova B, Morris KV (2010) Transcriptional gene silencing through epigenetic changes mediated by non-coding RNAs. Curr Opin Mol Ther 12: 214–222. 20373265 PMC2861437

[29] Caley DP, Pink RC, Trujillano D, Carter DRF (2010) Long noncoding RNAs, chromatin and development. Scient World J 10: 90–102. 10.1100/tsw.2010.7 PMC5763804 20062956

[30] Guttman M, Rinn JL (2012) Modular regulatory principles of large non-coding RNAs. Nature 482: 339–346. 22337053 10.1038/nature10887 PMC4197003

[31] Ulveling D, Francastel C, Hube F (2011) When one is better than two: RNA with dual functions. Biochimie 93: 633–644. 21111023 10.1016/j.biochi.2010.11.004

[32] Sawata M, Yoshino D, Takeuchi H, Kamikouchi A, Ohashi K, et al (2002) Identification and punctate nuclear localization of a novel noncoding RNA, Ks-1, from the honeybee brain. RNA 8: 772–785. 12088150 10.1017/s1355838202028790 PMC1370296

[33] Sawata M, Takeuchi H, Kubo T (2004) Identification and analysis of the minimal promoter activity of a novel noncoding nuclear RNA gene, AncR-1, from the honeybee (*Apis mellifera* L.). RNA 10: 1047–1058. 15208441 10.1261/rna.5231504 PMC1370596

[34] Woodard SH, Fischman BJ, Venkat A, Hudson ME, Varala K, et al (2011) Genes involved in convergent evolution of eusociality in bees. Proc Natl Acad Sci U S A 108: 7472–7477. 21482769 10.1073/pnas.1103457108 PMC3088614

[35] Linksvayer TA, Rueppell O, Siegel A, Kaftanoglu O, Page Jr RE, et al (2009) The genetic basis of transgressive ovary size in honeybee workers. Genetics 183: 693–707. 19620393 10.1534/genetics.109.105452 PMC2766328

[36] Batista PJ, Chang HY (2013) Long noncoding RNAs: cellular address codes in development and disease. Cell 152: 1298–1307. 23498938 10.1016/j.cell.2013.02.012 PMC3651923

[37] Kiya T, Ugajin A, Kunieda T, Kubo T (2012) Identification of *kakusei*, a nuclear non-coding RNA, as an immediate early gene from the honeybee, and its application for neuroethological study. Int J Mol Sci 13: 15496–15509. 23443077 10.3390/ijms131215496 PMC3546645

[38] Dinger ME, Pang KC, Mercer TR, Mattick JS (2008) Differentiating protein-coding and noncoding RNA: challenges and ambiguities. PLoS Comp Biol 4: e1000176. 10.1371/journal.pcbi.1000176 PMC2518207 19043537

[39] Weaver DB, Anzola JM, Evans JD, Reid JG, Reese JT, et al (2007) Computational and transcriptional evidence for microRNAs in the honey bee genome. Genome Biol 8: R97. 17543122 10.1186/gb-2007-8-6-r97 PMC2394756

[40] Behura SK, Whitfield CW (2010) Correlated expression patterns of microRNA genes with age-dependent behavioural changes in honeybee. Insect Mol Biol 19: 431–439. 20491979 10.1111/j.1365-2583.2010.01010.x

[41] Chen X, Yu X, Cai Y, Zheng H, Yu D, et al (2010) Next-generation small RNA sequencing for microRNAs profiling in the honey bee *Apis mellifera* . Insect Mol Biol 19: 799–805. 20807255 10.1111/j.1365-2583.2010.01039.x

[42] Jung C-H, Hansen MA, Makunin IV, Korbie DJ, Mattick JS (2010) Identification of novel non-coding RNAs using profiles of short sequence reads from next generation sequencing data. BMC Genomics 11: e77. 10.1186/1471-2164-11-77 PMC2825236 20113528

[43] Okazaki Y, Furuno M, Kasukawa T, Adachi J, Bono H, et al (2002) Analysis of the mouse transcriptome based on functional annotation of 60,770 full-length cDNAs. Nature 420: 563–573. 12466851 10.1038/nature01266

[44] Leontis NB, Lescoute A, Westhof E (2006) The building blocks and motifs of RNA architecture. Curr Opin Struct Biol 16: 279–287. 16713707 10.1016/j.sbi.2006.05.009 PMC4857889

[45] Colonello-Frattini NA, Hartfelder K (2009) Differential gene expression profiling in mucus glands of honey bee (*Apis mellifera*) drones during sexual maturation. Apidologie 40: 481–495.

[46] Liu F, Li W, Li Z, Zhang S, Chen S, et al (2011) High-abundance mRNAs in *Apis mellifera*: comparison between nurses and foragers. J Insect Physiol 57: 274–279. 21115016 10.1016/j.jinsphys.2010.11.015

[47] Nakaya HI, Amaral PP, Louro R, Lopes A, Fachel AA, et al (2007) Genome mapping and expression analyses of human intronic noncoding RNAs reveal tissue-specific patterns and enrichment in genes related to regulation of transcription. Genome Biol 8: R43. 17386095 10.1186/gb-2007-8-3-r43 PMC1868932

[48] Rao JP, Zafar RS, Sodja A (1988) Transcriptional activity at the 3′end of the actin gene at 5C on the X-chromosome of *Drosophila melanogaster*.. Biochim Biophys Acta 950: 30–44. 2896018 10.1016/0167-4781(88)90070-x

[49] Wu Q, Kim YC, Lu J, Xuan Z, Chen J, et al (2008) Poly A- transcripts expressed in HeLa cells. Plos One 3: e2803. 18665230 10.1371/journal.pone.0002803 PMC2481391

[50] Cawley S, Bekiranov S, Ng HH, Kapranov P, Sekinger EA, et al (2004) Unbiased mapping of transcription factor binding sites along human chromosomes 21 and 22 points to widespread regulation of noncoding RNAs. Cell 116: 499–509. 14980218 10.1016/s0092-8674(04)00127-8

[51] Louro R, Nakaya HI, Amaral PP, Festa F, Sogayar MC, et al (2007) Androgen responsive intronic non-coding RNAs. BMC Biology 5: e4. 10.1186/1741-7007-5-4 PMC1800835 17263875

[52] Mallik M, Lakhotia SC (2009) The developmentally active and stress-inducible noncoding hsr omega gene is a novel regulator of apoptosis in *Drosophila* . Genetics 183: 831–852. 19737742 10.1534/genetics.109.108571 PMC2778980

[53] Onorati MC, Lazzaro S, Mallik M, Ingrassia AMR, Carreca AP, et al (2011) The ISWI chromatin remodeler organizes the hsr omega ncRNA-containing omega speckle nuclear compartments. PLoS Genetics 7: e1002096. 21637796 10.1371/journal.pgen.1002096 PMC3102753

[54] Lakhotia SC (2012) Long non-coding RNAs coordinate cellular responses to stress. Wiley Interdisc Rev - RNA 3: 779–796. 10.1002/wrna.1135 22976942

[55] Azevedo SV, Martinez Caranton OA, de Oliveira TL, Hartfelder K (2011) Differential expression of hypoxia pathway genes in honey bee (*Apis mellifera* L.) caste development. J Insect Physiol 57: 38–45. 20887729 10.1016/j.jinsphys.2010.09.004

[56] Asencot M, Lensky Y (1985) The phagostimulatory effect of sugars on the induction of queenliness in female honeybee (*Apis mellifera* L.) larvae. Comp Biochem Physiol A 81: 203–208.

[57] Kucharski R, Maleszka J, Foret S, Maleszka R (2008) Nutritional control of reproductive status in honeybees via DNA methylation. Science 319: 1827–1830. 18339900 10.1126/science.1153069

[58] Büning J (1994) The Insect Ovary. London: Chapman & Hall. 400 p.

[59] Wayne ML, Hackett JB, Mackay TFC (1997) Quantitative genetics of ovariole number in *Drosophila melanogaster*.1. Segregating variation and fitness. Evolution 51: 1156–1163. 28565484 10.1111/j.1558-5646.1997.tb03963.x

[60] Godt D, Laski FA (1995) Mechanisms of cell rearangement and cell recruitment in *Drosophila* ovary morphogenesis and the requirement of *bric-a-brac*.. Development 121: 173–187. 7867498 10.1242/dev.121.1.173

[61] King FJ, Szakmary A, Cox DN, Lin HF (2001) Yb modulates the divisions of both germline and somatic stem cells through piwi- and hh-mediated mechanisms in the *Drosophila* ovary. Mol Cell 7: 497–508. 11463375 10.1016/s1097-2765(01)00197-6

[62] Sarikaya DP, Belay AA, Ahuja A, Dorta A, Green II DA, et al (2012) The roles of cell size and cell number in determining ovariole number in *Drosophila* . Dev Biol 363: 279–289. 22200592 10.1016/j.ydbio.2011.12.017

[63] Michelette ERD, Soares AEE (1993) Characterization of preimaginal developmental stages in Africanized honey bee workers (*Apis mellifera* L). Apidologie 24: 431–440.

[64] Durham AM, Kashiwabara AY, Matsunaga FTG, Ahagon PH, Rainone F, et al (2005) EGene: a configurable pipeline generation system for automated sequence analysis. Bioinformatics 21: 2812–2813. 15814554 10.1093/bioinformatics/bti424

[65] Huang XQ, Madan A (1999) CAP3: a DNA sequence assembly program. Genome Res 9: 868–877. 10508846 10.1101/gr.9.9.868 PMC310812

[66] Rutherford K, Parkhill J, Crook J, Horsnell T, Rice P, et al (2000) Artemis: sequence visualization and annotation. Bioinformatics 16: 944–945. 11120685 10.1093/bioinformatics/16.10.944

[67] Gruber AR, Lorenz R, Bernhart SH, Neuboeck R, Hofacker IL (2008) The Vienna RNA Websuite. Nucleic Acids Res 36: W70–W74. 18424795 10.1093/nar/gkn188 PMC2447809

[68] Fickett JW (1982) Recognition of protein coding regions in DNA-sequences. Nucleic Acids Res 10: 5303–5318. 7145702 10.1093/nar/10.17.5303 PMC320873

[69] Kong L, Zhang Y, Ye Z-Q, Liu X-Q, Zhao S-Q, et al (2007) CPC: assess the protein-coding potential of transcripts using sequence features and support vector machine. Nucleic Acids Res 35: W345–W349. 17631615 10.1093/nar/gkm391 PMC1933232

[70] Lourenço AP, Mackert A, Cristino AS, Simoes ZLP (2008) Validation of reference genes for gene expression studies in the honey bee, *Apis mellifera*, by quantitative real-time RT-PCR. Apidologie 39: 372–385.

[71] Livak KJ, Schmittgen TD (2001) Analysis of relative gene expression data using real-time quantitative PCR and the 2(T)(-Delta Delta C) method. Methods 25: 402–408. 11846609 10.1006/meth.2001.1262

[72] Saunders C, Cohen RS (1999) The role of oocyte transcription, the 5′UTR, and translation repression and derepression in *Drosophila gurken* mRNA and protein localization. Mol. Cell 3: 43–54. 10.1016/s1097-2765(00)80173-2 10024878

